# Editorial: Multi-omics studies on aging and age-related diseases

**DOI:** 10.3389/fcell.2024.1374424

**Published:** 2024-01-31

**Authors:** He-Ping Wang, Nathan Basisty, Jia-Hua Qu, Xiaoman Wang

**Affiliations:** ^1^ State Key Laboratory of Common Mechanism Research for Major Diseases, Department of Biochemistry and Molecular Biology, Institute of Basic Medical Sciences, Chinese Academy of Medical Sciences and Peking Union Medical College, Beijing, China; ^2^ Translational Gerontology Branch, National Institute on Aging, National Institutes of Health, Baltimore, MD, United States; ^3^ Department of Host-Microbe Interactions, St. Jude Children’s Research Hospital, Memphis, TN, United States

**Keywords:** multi-omics, aging, age-related diseases, biomarker, cancer, Alzheimer’s disease

Aging is a natural and inevitable process in all living organisms, characterized by the decline of physiological functions and increased susceptibility to age-related diseases (de Magalhães, 2013; Hou et al., 2019). These diseases often share common aging-related risk factors, such as chronic inflammation, oxidative stress, and impaired cellular repair mechanisms. Fueled by innovations in modern omic technologies and computational methods, scientists are able to quantify cellular senescence level (Wang et al., 2022) and profile very complex and heterogeneous age-related phenotypes with unprecedented molecular detail (Zhang et al., 2019; Qu et al., 2023). These insights will enhance our understanding of aging mechanisms and improve the precision and breadth of biomarker signatures, ultimately translating into therapeutic benefits to promote healthy aging. Multi-omic methods provide great opportunities to systematically reveal the critical molecular alterations of aging from a multidimensional perspective.

As the body is a complex system, studies at a singular level were not able to unravel the multiple levels of coordinated regulatory mechanisms (Qu et al., 2022), particularly those underlying the process of aging and development of age-related diseases. In this Research Topic, we aimed to showcase exemplar studies that leverage multi-omic technology to discover biomarkers of aging and aging-related diseases. Four papers on this Research Topic utilized genomic data, transcriptomic data, metabolomic data and proteomic data to identify potential biomarkers of aging and age-related diseases, including cancers and Alzheimer’s disease (AD), providing insight into potential therapeutic targets and anti-aging strategies.

The process of brain aging is an imbalance in intracellular homeostasis that impacts the functional capacities of brain (Mattson and Arumugam, 2018). However, the aging biomarkers and pathways in normal brain regions remain elusive. Xu et al. (López-Otín et al., 2023) addressed this Research Topic by leveraging public transcriptomics datasets. In a meta-analysis, the authors of this study found 94 common differentially expressed genes among three brain regions during aging. Consistent with previous studies, seven upregulated genes are enriched mainly in neuropeptide hormone activity. Conversely, downregulated genes were enriched with immune and inflammatory systems, suggesting that a declined inflammatory response could potentially be a key factor in promoting healthy brain aging. Furthermore, network analyses revealed that CD44, CD93 and CD163 were identified as biomarkers of natural brain aging, which are proposed as new molecules for potential estimation of brain aging in healthy populations.

The prevalence of cancer increases with age, highlighting the intimate connection between the aging process and cancer development9. Hu et al. dissected differences in tumor proliferation cycle phenotypes and examined their relationship with the tumor classification and clinical outcomes. Using the transcriptome of 691 eligible samples from The Cancer Genome Atlas (TCGA) and GEO database, Hu et al. recognized diverse proliferation cycle phenotypes within gastric cancer. The authors also analyzed tumor burden mutation from the TCGA cohort to explore the differences in genomic changes associated with these variations. Through LASSO regression method and Cox regression analysis, three genes GPC3, GPX3 and PRICKLE1 were identified as prognostic risk signatures for overall survival among gastric cancer patients, offering tailored diagnostic and treatment strategies for gastric cancer.

Untargeted metabolomics technology enables the exploration of connections between small molecule biomarkers and disease mechanisms. Although cerebrospinal fluid (CSF) biomarkers are widely used in predictive and prognostic effects of AD progression (Simrén et al., 2023), significant associations between the untargeted CSF metabolome and the panel of automated CSF immunoassays (i.e., NeuroToolKit (NTK) panel, containing established core AD biomarkers) have not been examined. Through metabolome-wide association study (MWAS), 269 CSF metabolites reflecting brain amyloidosis and tau pathology were associated with the NTK biomarkers. Mendelian randomization (MR) analysis determined causality between CSF metabolites and NTK biomarkers. Metabolites of soluble triggering receptors, such as palmitoyl sphingomyelin (d18:1/16:0) for myeloid cells 2 (sTREM2) and erythritol for amyloid β (Aβ40) and α-synuclein, were identified associated with AD-related pathology (Dong et al.), advancing our knowledge of AD-related causality from metabolomic perspective.

Translated variant proteins or polypeptide products ultimately serve as the functional units associated with diseases (Reilly et al.). The recent integration of genomics and mass spectrometry-based proteomics for biomarker discovery, known as proteogenomics, has significantly advanced our understanding of disease risk variants, precision medicine, and biomarker identification. Reilly et al. reviewed proteogenomics applications in biomarker discovery on cancers and neurodegenerative diseases. Reilly et al. summarized emerging proteogenomic methods for biomarker discovery, and proposed a comprehensive proteogenomic strategy in disease diagnosis and patient stratification for age-related diseases.

We express our gratitude to all contributors who have chosen this Research Topic for their outstanding scientific contributions. In addition to cancers and AD that the four articles in this Research Topic focused on, other aging processes and age-related pathologies, such as metabolic syndrome, cardiovascular diseases, frailty and others, also benefit from the development and application of multi-omic techniques (Zheng et al., 2022; Sahu et al., 2023; Zhang et al., 2023). For instance, the methods used and concepts proposed in these articles in this Research Topic can be applied in studying other age-relate diseases. It is our hope that this Research Topic will significantly advance the field, uncovering novel insights in multi-omics studies on aging and age-related disease.
